# Similarity of Recombinant Human Perlecan Domain 1 by Alternative Expression Systems Bioactive Heterogenous Recombinant Human Perlecan D1

**DOI:** 10.1186/1472-6750-10-66

**Published:** 2010-09-09

**Authors:** April L Ellis, Wensheng Pan, Guang Yang, Kim Jones, Christine Chuang, John M Whitelock, Arthur A DeCarlo

**Affiliations:** 1 Agenta Biotechnologies, Inc., Innovation Depot, 1500 1st Ave. N., Birmingham, AL 35203, USA; 2 Southern Research Institute, Birmingham, AL, 35205, USA; 3 University of Alabama at Birmingham, Birmingham, AL 35294, USA; 4 Graduate School of Biomedical Engineering, University of New South Wales, Sydney, Australia

## Abstract

**Background:**

Heparan sulfate glycosaminoglycans are diverse components of certain proteoglycans and are known to interact with growth factors as a co-receptor necessary to induce signalling and growth factor activity. In this report we characterize heterogeneously glycosylated recombinant human perlecan domain 1 (HSPG2 abbreviated as rhPln.D1) synthesized in either HEK 293 cells or HUVECs by transient gene delivery using either adenoviral or expression plasmid technology.

**Results:**

By SDS-PAGE analysis following anion exchange chromatography, the recombinant proteoglycans appeared to possess glycosaminoglycan chains ranging, in total, from 6 kDa to >90 kDa per recombinant. Immunoblot analysis of enzyme-digested high M_r _rhPln.D1 demonstrated that the rhPln.D1 was synthesized as either a chondroitin sulfate or heparan sulfate proteoglycan, in an approximately 2:1 ratio, with negligible hybrids. Secondary structure analysis suggested helices and sheets in both recombinant species. rhPln.D1 demonstrated binding to rhFGF-2 with an apparent k_D _of 2 ± 0.2 nM with almost complete susceptibility to digestion by heparinase III in ligand blot analysis but not to chondroitinase digestion. Additionally, we demonstrate HS-mediated binding of both rhPln.D1 species to several other GFs. Finally, we corroborate the augmentation of FGF-mediated cell activation by rhPln.D1 and demonstrate mitogenic signalling through the FGFR1c receptor.

**Conclusions:**

With importance especially to the emerging field of DNA-based therapeutics, we have shown here that proteoglycan synthesis, in different cell lines where GAG profiles typically differ, can be directed by recombinant technology to produce populations of bioactive recombinants with highly similar GAG profiles.

## Background

Heparan sulfate glycosaminoglycans (GAGs) that decorate proteoglycan core proteins vary in chain length, net charge, and charge distribution, making them quite heterogenous. This is due, in large part, to post-translational addition, then removal, of sulfate groups to various positions on the constituent monosaccharides, orchestrated by a battery of enzymes in a tissue- or cell-specific manner [1]. This level of complexity and potential regulation afforded by the proteoglycan system can, therefore, be considered relatively high.

The GAG heparan sulfate has an important role in regulating cell function by serving as a co-factor, or co-receptor, in growth-factor (GF) interactions. Indeed, without available heparan sulfate proteoglycans (HSPGs), growth factor activity would be limited; during development, heparan sulfate (HS) deficiency is lethal [2] while targeted disruption of normal HS post translational modification results in a structurally altered HS and lethality, with kidney, lung, and skeletal defects [3,4].

The most highly studied and well-understood of the interactions between HSPGs and GFs is that between heparan sulfate and fibroblast growth factors 1 and 2 (FGF-1, FGF-2), two closely related members of a large FGF family [5]. FGFs are mitogenic for a variety of cell types, promoting differentiation and wound healing [6]. The crystal structure of the ligand/receptor complex for FGF-2 and the FGF receptor (PDB 1FQ9) has been determined, clarifying the importance of heparin (or heparan sulfate) as a co-receptor in dimerization of the growth factors and their receptors leading to activation [7].

Native perlecan (HSPG2, abbreviated here as Pln) is an extracellular matrix proteoglycan with three HS side chains linked to a large core protein of approximately 450 kDa in 5 recognizable domains which, themselves, are comprised of subdomains [8,9]. Domain 1 of perlecan (Pln.D1) comprises only a small fraction of the native perlecan encoding an approximately 21 kDa protein core with the primary sequence potential for three O-linked GAG chains. While the C-terminus of Pln.D1 is known to have sequence homology with an SEA module that is also found in a related HSPG, agrin, and in regions of other biomolecules receiving extensive O-linked glycosylation [10], the tertiary structure of Pln.D1 is not known.

The HS of Pln.D1 is reported to promote cell adhesion [11-14], to promote proliferation and/or differentiation [15], and to bind and deliver growth factors [11,16-19]. Independently, there is strong scientific support and rationale for a role of perlecan in angiogenesis or neovascularization [17,20,21]. Interference between FGF or VEGF and perlecan HS binding has been shown to diminish the functional activity of these GF's [6,22].

Numerous growth factors are being tested as therapeutic agents [23-29] and some are currently commercially available. It is reasonable, therefore, that increased levels of soluble HS proteoglycan delivered concomitantly with certain growth factors would lead to enhanced growth factor activity in a physiological manner and be of value in wound management and healing.

While recombinant mouse [30] and human [31-33] perlecan D1 have been partially characterized, here we provide characterization of two human Pln.D1 recombinants generated from adenovirus or plasmid DNA expression.

## Methods

### Construction of Pln.D1 transgene

Perlecan D1 (Pln.D1) was cloned by reverse-transcriptase extension of total human endothelial cell RNA using perlecan specific primers, then amplified by PCR and cloned into pcDNA 3.3+ (Invitrogen) incorporating amino acids 1-198 or 1-247 (to include the region between domains 1 and 2 up to and including the first amino acid of domain 2). In each clone a Kozac-like sequence (ccacc) was strengthened by inserting cca immediately 5' to the native ccatg at -2 relative to the start codon (atg). The native secretory leader sequence was not modified. A 6×-His tag was added to the C-terminus of each clone for identification. Sequence identity of the full-length domain 1 clone compared to the HSPG2 (reference NP_005520.4) was confirmed. DNA stocks were prepared with Qiagen endonuclease free MaxiPrep kits.

### Construction of Pln.D1 adenovirus

Pln.198 or Pln.247 transgenes were subsequently cloned into an adenoviral expression construct with a CMV-5 promoter (Adenovator, Qbiogene). Adenovirus with a Pln.D1 transgene (Pln.198-Ad and Pln.247-Ad) were isolated using a ViraKit AdenoMini-24 system (ViraPur).

### Synthesis of rhPln.D1

Hek 293 cells were grown on 100 mm culture dishes in Dulbecco's Modified Eagles Medium (DMEM, Sigma Chemical Co.) with 44 mM NaHCO3, pH 7.2, and supplemented with 10% (v/v) fetal bovine serum (FBS), 100 U/ml penicillin and 0.1 mg/ml streptomycin (Pen/Strep) at 37°C with 5% CO_2 _in a humidified incubation chamber. HEK 293 cells were cultured to 90% confluence then transfected with lipoplexes containing Pln.198-pcDNA3.3+ or Pln.247-pcDNA3.3+ and lipofectamine2000 (LF2K, Life Technologies) in 1:1.5 or 1:2 μg DNA: μg LF2K standard ratio and following the manufacturer's instructions in the presence of serum. Cells were incubated for 3 days following transfection and conditioned media containing secreted protein was collected.

Alternatively, HEK 293 cells cultured to 90% confluency were infected with Pln.198-Ad or Pln.247-Ad at TCID50 1 × 10^7^/ml and incubated for 5 days to express protein.

Conditioned media containing secreted protein synthesized by either method was collected and separated from cellular debris by centrifugation for 10 min at 5K rpm and 4°C and then filtered through a 0.2 μm filter prior to anionic exchange chromatography.

### Purification of high M_r _rhPln.D1

Filtered media was diluted 10× into Binding Buffer (containing 20 mM Tris, 62.5 mM NaCl, 0.25% Tween-20, 15 mM sodium azide, pH 8.0) and bound to a Hitrap anion exchange column (Amersham) connected to an AktaPrime FPLC at 5 ml/min. The column was washed to a flat baseline, without Tween-20, and bound contaminants were removed by a continual wash with 10% Elution Buffer (20 mM Tris, 1 M NaCl, 15 mM sodium azide, pH 8.0) until a flat baseline was reached. Remaining bound rhPln.D1 was eluted from the column with a 50 ml gradient to 100% Elution Buffer at 2.5 ml/min collecting 1 ml fractions and monitoring absorbance at 280 nm.

Analyzing the fractions by techniques described below, fractions containing the high molecular weight (M_r_), glycosylated protein were pooled and the buffer was exchanged to 50 mM HEPES, 250 mM NaCl, pH 8.0 by repeated dilution and concentration using 6 ml centricones (Vivascience, Sartouris Stedim) with 10 kDa MWCO. After anionic exchange chromatography and pooling of heterogeneous fractions, enriched high M_r _rhPln.247 from 100 ml conditioned medium generated by adenovirus yielded 25 ml of 140 μg/ml protein as determined by Abs_280 _using a calculated extinction coefficient of 28795 M^-1^cm^-1 ^for the recombinant. Enriched high M_r _rhPln.198 from 100 ml conditioned medium yielded 10 ml of 140 ug/ml as determined by Abs_280 _and a calculated extinction coefficient of 25440 M^-1 ^cm^-1^. Plasmid transfection generated approximately 3 fold less recombinant than adenoviral infection, per volume conditioned media. For storage, 1% glycerol was added to the protein stock and protein was stored at -20°C.

### Western blot

Proteins were separated on a 12% SDS-PAGE gel at 200 V and transferred to nitrocellulose utilizing a BioRad Mini Transblot system at 0.3 Amp for 90 mins. The blot was blocked in 1.3 mM KCl, 0.8 mM KH_2_PO_4_, 68 mM NaCl, and 4.1 mM Na_2_HPO_4 _(PBS) containing 15 mM sodium azide and 0.1% Tween 20 (PBS-Tween) for 30 min at room temperature and with agitation. The blot was then incubated in primary antibody CSI 001-71 (Assay Designs, clone A71) overnight at 4°C with rocking, washed in PBS-Tween, and incubated in goat-anti-mouse AP conjugated secondary antibody (Abcam) for 2 hr at 4°C with rocking. After washing, the blot was developed with SigmaFast BCIP/NBT tablets dissolved in _dd_H_2_O and imaged on a Kodak GelLogic 1500 Imaging System.

### Stains-All/Silver

Acidic and/or glycosylated Pln.D1 was visualized by SDS-PAGE and Stains-All staining enhanced by silver nitrate similar to the protocols of Goldberg and Warner [34]. Proteins were separated on a 12% SDS-PAGE gel, and the gel was washed for 30 mins in 100 ml of 25% isopropanol with 5 solution changes to remove the SDS. The gel was then incubated in 30 ml of Stains-All staining solution (0.025% Stains-All in 30 mM Tris, 7.5% formamide, 25% isopropanol, pH 8.0 from a stock of 0.25% Stains-All in formamide) overnight in a light-tight box at room temperature with agitation. The stain was removed, and the gel was destained in 100 ml of 25% isopropanol maintaining light tightness until the background was a light pink and protein bands could be discerned. The gel was imaged on a Kodak GelLogic 1500 with transmitted white light for 2.4 ms exposure with an f-stop of 5.6. After imaging, the gel was allowed to fade by light in 25% isopropanol intermittently exchanged with _dd_H_2_O to a clear background. The gel was then washed thrice with _dd_H_2_O and incubated in 60 ml of freshly prepared 12 mM AgNO3 for 30 mins, rinsed three times in _dd_H_2_O, and developed with 70 ml of 0.28 M Na_2_HCO_3_, 0.15% formaldehyde after rinsing the gel twice quickly with approximately 15 ml of the same solution. The development reaction was stopped with 10% acetic acid. The gel was again imaged using the GelLogic 1500.

### ELISA

Enzyme linked immunosorbant assays (ELISA) were performed in polystyrene microtiter wells. Proteins were coated overnight at 4°C onto the surfaces in PBS and 15 mM sodium azide (PBS-N__3__). All wells were then blocked and washed in PBS-Tween. Primary murine antibodies were applied in PBS-Tween at a concentration of 0.5 μg/ml overnight at 4°C. Secondary goat anti-mouse antibodies conjugated with alkaline phosphatase (Abcam) were applied at a concentration of 1 μg/ml for 2 h then AP activity was monitored at 405 nm by hydrolysis of the substrate 4-Nitrophenylphosphate (Boehringer Mannheim, Germany) in 10 mM Tris, pH 9.5 with 5 mM MgCl_2 _using a Bio-Tek Instruments, Inc. μQuant spectrophotometer (absorbance maximum of 3.0 ELISA units). Apparent dissociation constants (k_D_) were derived by solid-phase with ELISA as previously described [35] and presented with standard deviation.

### Mass spectrometry analysis

MALDI-TOF mass spectrometry was performed after tryptic digestion of rhPln.198 and rhPln.247 purified protein. Enzymatic digestion with trypsin (12.5 ng/μl) (Promega Gold Trypsin Mass Spectrometry Grade) was carried out for sixteen hours at 37°C. Peptide solutions were then extracted using two washes of 100 μl of a 50/50 solution of 5% formic acid and acetonitrile for thirty minutes. Supernatants were collected and dried down in a Savant SpeedVac. Samples were resuspended in 40 μl of 0.1% formic acid. C^18 ^ZipTips (Millipore) were used to desalt peptide mixtures before analysis. An aliquot (5-10 μl) of each digest was loaded onto a 5 mm × 100 μm i.d. C_18 _reverse-phase cartridge at 2 μl/min. After washing the cartridge for 5 min with 0.1% formic acid in _dd_H_2_0, the bound peptides were flushed onto a 22 cm × 100 μm i.d. C_18 _reverse-phase analytical column with a 25 min linear 5-50% acetonitrile gradient in 0.1% formic acid at 500 nl/min. The column was washed with 90% acetonitrile-0.1% formic acid for 15 min and then re-equilibrated with 5% acetonitrile-0.1% formic acid for 24 min. The eluted peptides were passed directly from the tip into a modified MicroIonSpray interface of an Applied Biosystems-MDS-Sciex (Concorde, Ontario, Canada) 4000 Qtrap mass spectrometer. Eluted peptides were subjected to a survey MS scan to determine the top three most intense ions. A second scan (the enhanced resolution scan) determined the charge state of the selected ions. Finally, enhanced product ion scans were carried out to obtain the tandem mass spectrum of the selected parent ions (with the declustering potential raised to 100 V) over the range from *m/z *400-1500. Spectra were centroided and de-isotoped by Analyst Software, version 1.42 (Applied Biosystems). These tandem mass spectrometry data were processed to provide protein identifications using an in-house MASCOT search engine (Matrix Science) against the NCBInr Human Protein database.

### Circular dichroism of rhPln.D1

CD was performed on 5.6 μM rhPln.198 or 8.4 μM rhPln.247 in 25 mM HEPES, 100 mM NaCl, pH 7.4 using a Jasco J-815 spectrometer at 20°C, 0.5 mm pathlength cell, 4 accumulations, 1 nm steps, and from 240 to 190 nm. Buffer was the baseline and was subtracted from the protein spectrum. All spectra were computationally smoothed and filtered (FFT) with the Jasco Spectra Analysis software. Estimations of helix and strand content were calculated with Raussens method http://perry.freeshell.org/raussens.html.

### Growth factor ligand blot

rhPln.D1 samples were separated on a 12% polyacrylamide gel and transferred to nitrocellulose for 1.5 hr at 0.3 Amp. After blocking, blots were incubated in 0.1 μg/ml rhFGF-2 (R&D Systems) in PBS-Tween overnight at 4°C with rocking. Blots were washed in PBS-Tween and incubated in 1 μg/ml anti-FGF-biotin antibody (R&D Systems) for 4 hr at 4°C with rocking, then washed in PBS-Tween, and incubated in 1 μg/ml streptavidin-alkaline phosphatase (R&D Systems) for 2 hr at 4°C with rocking. Blots were then developed and imaged as described in the section *Western blots *above.

### Glycosidase digestion

Enriched Pln.D1 was digested with heparinase I, II (Sigma or IBEX Technologies), and III (Seikagaku) and with chondroitinase ABC (Seikagaku) overnight at 37°C with 1 mU of enzyme/μg Pln.D1 in 50 mM HEPES, 200 mM NaCl, pH 7.4. Controls of undigested protein were also incubated in the same manner. Digested samples were examined by mAb 001-71 Western blot and Stains-All/Silver SDS-PAGE applying 2.8 μg protein/lane.

### Ligand binding ELISA

The ligand binding assay was a variant of the ELISA in which dilutions of ligand (rhFGF-2) were allowed to bind to a ligand binding protein such as rhPln.D1 that had been coated onto the wells in PBS-N_3_. The bound ligand was then detected with biotin-conjugated mAb specific for hFGF-2 which was followed by a streptavidin-AP conjugate and developed as described for ELISA. Apparent k_D _were derived as previously described [35] in these assays using serial dilutions of ligand with even amounts of coated ligand binding protein. Results are accompanied by standard deviation.

### Growth factor dot blots

Growth factors rhFGF-2, rhVEGF165, rhVEGF189, rhPDGF-BB, rhEGF, rhIGF, rhFGF-7, rhBMP-2, rhBMP-6, rhBMP-7, rhBMP-9, and rhBMP-14 were bound to nitrocellulose at 500 ng utilizing a BioRad dot blot apparatus. Blots were blocked in PBS-Tween and incubated in 1 μg/ml rhPln.198 or rhPln.247 (separate blots) in PBS-Tween overnight at 4°C with rocking. Alternatively, some blots were incubated in heparinise III digested rhPln.247 (also 1 μg/ml) overnight at 4°C with rocking. After washing, blots were incubated in 3 ug/ml rhPln.D1 primary antibody CSI 001-71 in PBS-Tween overnight at 4°C with rocking. Blots were then washed and incubated in 1 ug/ml goat-anti-mouse AP conjugated secondary antibody for 2 hr at 4°C with rocking. Blots were then developed and imaged as described.

### Baf32 proliferation assays

Proliferation of FGFR1c-expressing Baf32 cells were monitored via MTS reagent and absorbance at 490 nm with the addition of 0.3 nM rhFGF-2 and titration of rhPln.247. Baf32 cells were maintained in RPMI 1640 medium containing 10% v/v FBS, 10% v/v WEHI-3BD conditioned medium and 1% v/v penicillin/streptomycin. For the mitogenic assays, the Baf32 cells were transferred into IL-3 depleted medium in low serum for 24 h prior to experimentation and seeded into 96-well plates at a density of 2 × 10^4 ^cells/well in the presence of FGF-2 (0.03 nM), heparin (30 nM) and either HCAEC perlecan (2 μg/ml) or rhPln.247 (1-20 μg/ml). Cells were incubated for 96 h in 5% CO_2 _at 37°C and the amount of cells present was assessed using the MTS reagent (Promega, Madison, Wisconsin, USA) by adding to the cell cultures for 6 h prior to measuring the absorbance at 490 nm.

### HUVEC proliferation assays

HUVEC were purchased from Lifeline Cell Technologies, Inc. and cultured according to the manufacturer's instructions with LCT VascuLife EnGS medium including all supplied LifeFactors (2% serum, L-glutamine, Hydrocortisone, Heparin, Ascorbic acid, EGF, EnGs). For proliferation studies, low passage number cells (below P14) were seeded in 96 well plates directly into experimental medium at 10^4 ^cells/well in 200 μl Assay Medium, LCT VascuLife EnGs containing only selected LifeFactors (2% serum, L-glutamine, Hydrocortisone, EnGS). Experimental conditions included 5 ng/ml of growth factor (rhFGF-2, R&D Systems) in Assay Medium with dilutions of enriched rhPln.198 or rhPln.247. Cell count was measured after 48 hr incubation by conversion of Dojindo's CCK8 dye with 1 hr incubation on live cells. Resulting absorbance of the converted substrate was measured at 450 nm in a parallel microtiter plate. Measurements were adjusted for background with measurements taken from control media.

## Results

### Anion exchange chromatographic separation of rhPln.D1 by GAG level

**Figure 1:**
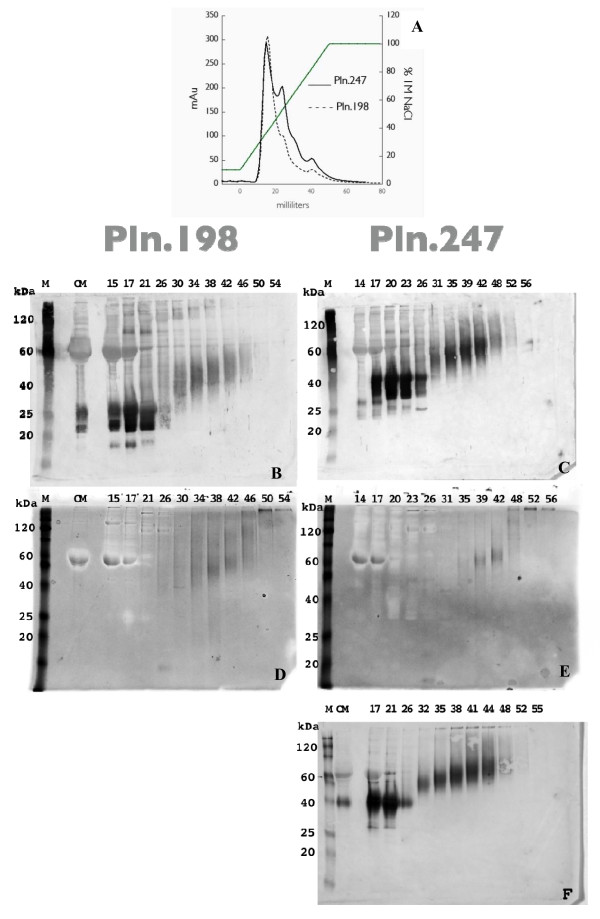
**Anion Exchange Chromatography of rhPln.247 conditioned media (CM)**. A: Chromatogram of elution profile for rhPln.198-Ad and rhPln.247-Ad CM expressed from HEK 293 cells; B and C: Western blots of anion exchange fractions with mAb CSI 001-71 recognizing the Pln.D1 core; D and E: Stains-All analysis of HEK 293 fractions; F: Fractions from HUVEC synthesis. Panels B and D represent rhPln.198 fractionation; panels C, E, F represent rhPln.247 fractionation. (M) Markers; (CM) Conditioned medium undiluted from cells. Similar data were generated using plasmid expression of pln.247 (not shown).

HEK 293 cells were infected with the Pln.247-Ad or the Pln.198-Ad and the conditioned medium was subjected to anion exchange chromatography with a linear NaCl gradient to 1 M (Figure 1A). Testing the hypothesis that retention on the anionic exchange column through increasing ionic strength of the elution buffer would be characterized by an immunopositive rhPln.D1 with increasing GAG chain length, we sampled a broad range of immunopositive fractions by Western blot using the anti-Pln.D1 mAb CSI 001-071 (Figure 1B,C). In the eluant, we found that the immunoreactive species with the lower net apparent kDa (shorter GAGs) eluted earliest (up to 26 ml) and that increasing salt eluted rhPln.D1 species with increasing GAG length and greater apparent kDa. The early fractions of each recombinant were characterized by highly immunoreactive, minimally glycosylated species, the lowest approximately where the unglycosylated core of Pln.198 (Figure 1B) and Pln.247 (Figure 1C) was expected to migrate. Beyond 30 ml, eluting from approximately 0.5 M NaCl, these lower M_r _species were absent while a broad range of immunoreactivity spanning 30-60 kDa upward (Pln.198) or 50-120 (Pln.247) eluted, gradually increasing in apparent M_r _through 52 ml (eluting in 1 M NaCl). The range of apparent kDa in these higher M_r _fractions suggested a recombinant species with GAG chains totalling 24 kDa to >90 kDa in rhPln.247, or average individual chain sizes of 8-30 kDa, assuming glycosylation of the 3 SGD linkage sites within rhPln.247. The higher M_r _fractions of Pln.198 did not appear to be glycosylated as extensively, with estimated GAG chains of 3-13 kDa again assuming glycosylation at each of the three SGD regions in rhPln.198. The synthesis of rhPln.247 in HUVEC culture resulted in matching data (Figure 1F, Western blot). Similar data were also produced when the HEK 293 cells were transfected instead with a mammalian expression plasmid encoding the Pln.247 transgene (data not shown).

While it appeared by immunoblot with mAb 001-71 that the overwhelming majority of both rhPln.198 and rhPln.247 were poorly glycosylated, the use of protein staining in companion Stains-All gels (Figure 1D,E) or silver stain (not shown) demonstrated that the more extensively glycosylated species constituted the majority of recombinant in the eluant, suggesting that the CSI 001-71 mAb had been more reactive with rhPln.D1 species having undeveloped or shortened GAG chains.

### Native Pln not detected in high M_r _rhPln.D1 preparation

**Figure 2:**
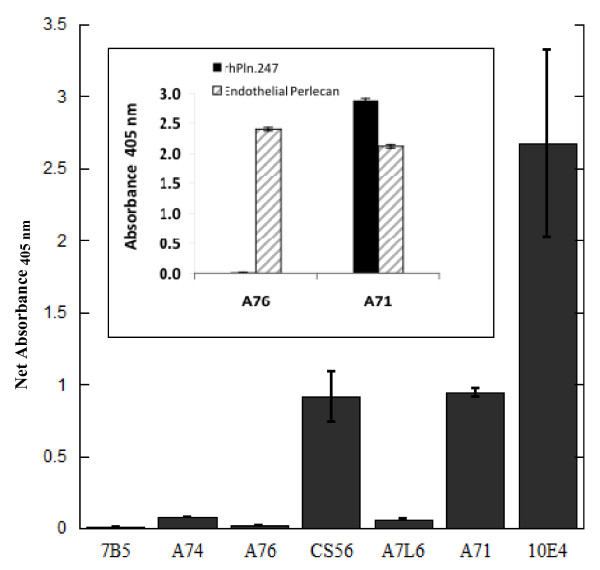
**ELISA identifies only Pln.D1 but not Pln.D3-5 in enriched rhPln.198**. The enriched high M_r _rhPln.198 synthesized by HEK 293 cells was used to coat microtiter wells that were subsequently blocked then incubated with 1.7 μg/ml of different primary antibodies as depicted in the legend. ELISA was completed as described in Methods. Background signal for each antibody binding to uncoated wells was subtracted to produce the net signal. Inset: ELISA data showing negligible mAb A76 reactivity against rhPln.247 and strong reactivity with full-length endothelial perlecan (purified as previously described [53]), while mAb A71 reacted strongly to both the native perlecan and the truncated recombinant. Net absorbance minus buffer-coated wells represented.

To support the hypothesis that the CSI-001-71 immunoreactive species eluting in relatively high salt were recombinant and not native Pln.D1, we pooled the fractions from 34-52 ml containing only high M_r _rhPln.D1, reduced the salt by buffer exchange, then aliquots were immobilized in microtiter wells and ELISA performed with a panel of anti-perlecan antibodies (Figure 2). These data indicated that the perlecan core was recognized only by the anti-D1 core antibody CSI 001-71 but not by the anti-D3 antibody 7B5 (Invitrogen cat. 13-4400) nor the anti-D4 antibody A7L6 (Millipore mAb1948) nor the anti-D5 antibody CSI 001-74. The recombinant pools were each found to have CS glycosylation recognized by mAb CS56 (Sigma Aldrich) while mAb 10E4 recognized the predominately unsulfated HS epitopes [36]. Interestingly, mAb CSI 001-76 (clone A76), that is known to recognize domain 1 of native perlecan (Figure 2 inset, endothelial perlecan), poorly recognized the truncated recombinants rhPln.198 (Figure 2) and rhPln.247 (inset, Figure 2) relative to mAb CSI 001-71 (A71).

**Figure 3:**
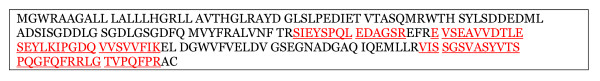
**Tryptic peptides of purified rhPln.198 by mass spectrometry**. Purified rhPln.198 was subjected to trypsin digest and fragments were analyzed by mass spectrometry. Red underline signifies fragments identified in this analysis of enriched rhPln.198.

Mass spectrometry analysis of trypsin-digested purified rhPln.198 resulted in the identification of several Pln.D1 fragments (Figure 3) but no other perlecan domains were detected. However, analysis of western blots with gels developed with silver or Stains-All suggested that the higher M_r _fractions selected for pooling and analysis had minor levels of contaminating proteins (Figure 1). By mass spectrometry of enriched rhPln.198 or rhPln.247, only three other minor protein contaminants were identified, presumably from the use of fetal bovine serum in the growth medium: bovine lumican, bovine hemoglobin, and BSA (data not shown).

### Circular dichroism

**Figure 4:**
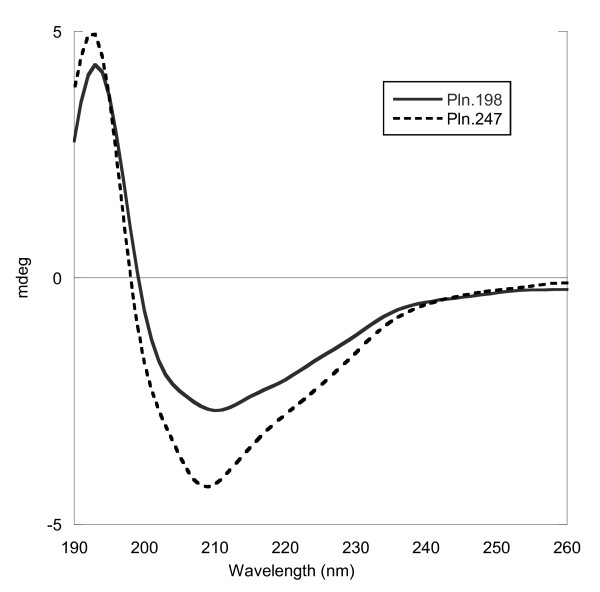
**Circular dichroism of 5.6 μM Pln.198 and 8.4 μM Pln.247 in 25 mM HEPES, 100 mM NaCl, pH 7.4 with baseline subtraction and FFT filter**. The differences in the spectra suggest a structural difference in the two proteins, likely due to the addition of the 47 amino acids on Pln.247 that form a cysteine knot.

The CD spectra of the Pln.198 and Pln.247 differ slightly (Figure 4), likely due to the probable cysteine knot structure at the C-terminus of Pln.247. Analysis of the spectra by Raussens method predicts Pln.198 to be 35.2% helical, 19.1% strand, 31.8% coil, and 12.5% turn, and Pln.247 to be 27.5% helix, 23.7% strand, 34.3% random coil, and 12.5% turn.

### CS and HS decorate rhPln.D1 but not together

**Figure 5:**
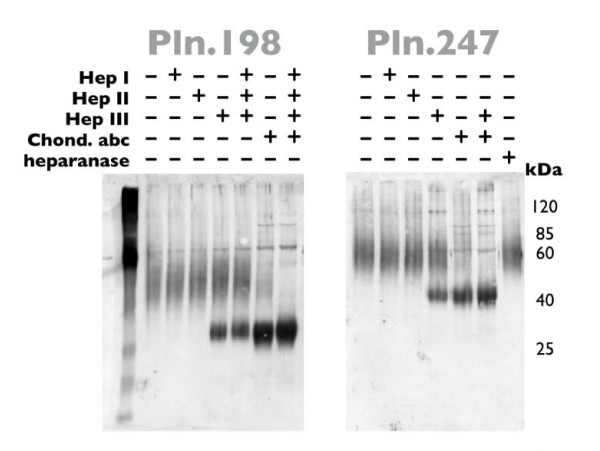
**Western blots of rhPln.D1 glycosidase digests**. Pooled anionic exchange fractions (34-52 ml) from HEK 293 culture (Pln.198) or HUVECs (Pln.247) were subjected to digestion with either buffer only, heparinase I (Hep I), heparinase II (Hep II), heparinase III (Hep III), chondroitinase ABC (Chond. abc), all three heparinases plus chondroitinase ABC, or heparanase. Treated samples were analyzed by western blot using mAb CSI 001-71. Similar data were generated using plasmid expression of pln.247 (not shown).

To assess the relative contributions of CS and HS to each of the recombinants, aliquots of the high M_r _rhPln.D1 preparations were digested with heparinases I, II, or III, or with chondroitinase ABC, or with mammalian heparanase (Figure 5). By western blot, treatment with heparinase I or II produced no measurable change in the staining pattern in either of the recombinant pools (rhPln.198 from HEK 293 cells and rhPln.247 from HUVEC). Treatment with heparinase III resulted in the appearance of low M_r _species similar to those seen early in the elution profile (Figure 1). Treatment with chondroitinase ABC resulted in significant loss of the major Pln.D1 immunoreactivity and a more dense low M_r _band. Treatment of the recombinant proteoglycans with the combination of heparinases and chondroitinase ABC resulted in further elimination of the higher M_r _immunoreactivity, and greater density in the low M_r _band. Treatment with mammalian heparanase did not demonstrate a clearly observable specific activity against the recombinant pools by this assay (Pln.247, Figure 5; Pln.198, data not shown). In summary, data from these experiments suggested that recombinant synthesis of different Pln.D1 variants in these two different cell types generated similar HS and CS levels. Also, it is important to note that digestion with either heparinase III or chondroitinase ABC did not produce intermediate bands of immunoreactivity or silver staining between the high M_r _rhPln.D1 and the dominant digest products (43 kDa, Pln.247; 33 kDa, Pln.198); this suggested that the core proteins were either fully decorated with HS (approximately 1/3 of total) or fully decorated with CS (approximately 2/3 of total), and that there were negligible hybrids.

### FGF-2 binding is mediated by rhPln.D1 HS but not CS

**Figure 6:**
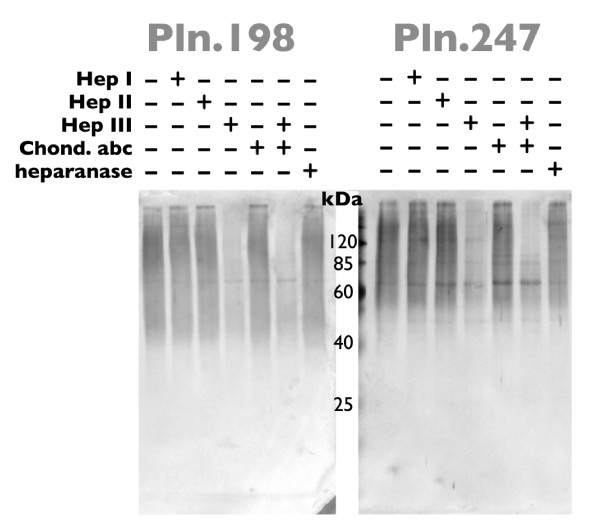
**FGF-2 ligand blot binding of glycosidase-digested rhPln.D1**. Pooled anionic exchange fractions of rhPln.198 or rhPln.247 from HEK 293 cell culture were subjected to digestion as designated; buffer only, heparinase I (Hep I), heparinase II (Hep II), heparinase III (Hep III), chondroitinase ABC (Chond. abc), all three heparinases plus chondroitinase ABC, or heparanase. Samples were subjected to SDS-PAGE and blotted to nitrocellulose. After blocking, the blot was then incubated with rhFGF-2 overnight, washed, then the bound rhFGF-2 was detected by anti-FGF-2 biotin-conjugates and streptavidin-alkaline phosphatase staining.

Knowing that a significant proportion of the rhPln.198 and rhPln.247 was glycosylated with HS, we tested the hypothesis that the rhPln.D1 would bind FGF-2. Applying rhFGF-2 in solution to the electrophoretically separated recombinant pools that had been immobilized on a nitrocellulose blot (Figure 6), the FGF-2 bound specifically to the rhPln.198 and rhPln.247. Further, digestion with heparinase III, but not chondroitinase ABC, mostly eliminated FGF binding to the enriched rhPln.D1 in this system.

**Figure 7:**
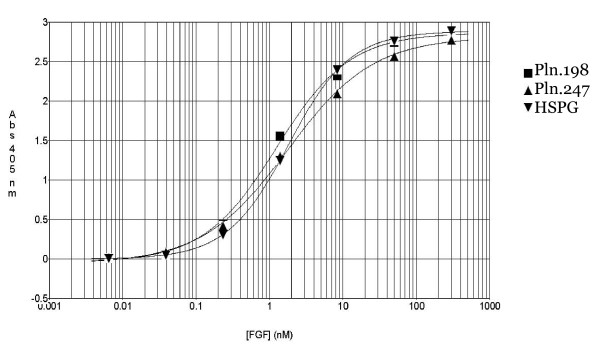
**Growth-factor binding to rhPln.198, rhPln.247, and HSPG in solid phase**. Commercial HSPG, Pln.247, or rhPln.198 were bound directly to polystyrene microtiter wells. The proteoglycan samples were incubated with 6-fold dilutions of rhFGF-2. After washing away unbound FGF, the bound FGF was detected with anti-FGF biotin conjugates.

Solid phase binding analysis of rhFGF-2 to the rhPln.198 and rhPln.247 demonstrated an estimated k_D _of 2.0 ± 0.2 nM for each, which was similar to binding of rhFGF-2 with a commercial HSPG (Sigma Aldrich) (Figure 7). Binding was the same when capturing the rhPln.D1 in the high M_r _preparations onto a solid-phase anti-perlecan mAb 001-71 ensuring that binding of FGF-2 to the enriched high M_r _Pln.D1 was a result of binding to the rhPln.198 and not a contaminant (data not shown).

### rhPln.D1 HS mediates binding to several growth factors

**Figure 8:**
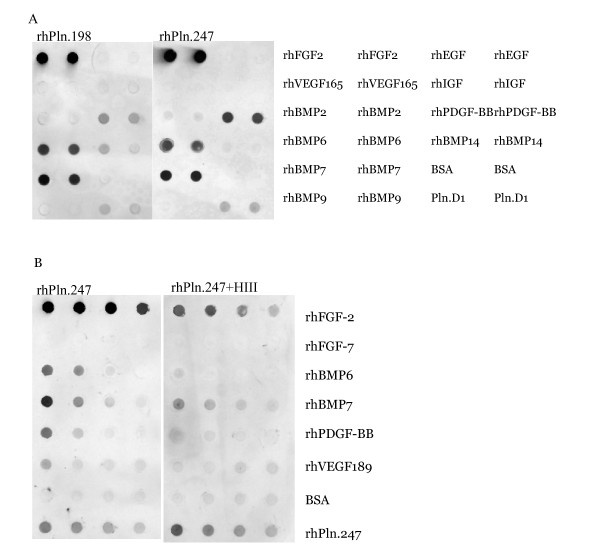
**Immunoblot analysis of growth factor binding to Pln.198 and Pln.247**. 500 ng growth factors were bound to nitrocellulose, the blots were blocked, and then incubated in either 1 ug/ml Pln.198 or Pln.247 (panel A) or rhPln.247 pre-digested with heparinase III (panel B). Detection was performed with anti-perlecan domain 1 primary antibody CSI 001-71. Note that while both the background BSA signal and the internal control rhPln.247 signals are slightly higher in panel B (bottom two samples), a decrease in the growth factor binding to Pln.247 was present after digestion.

Nitrocellulose immobilization of several growth factors and immunoblot analysis confirmed binding of rhPln.198 and rhPln.247 with the growth factors rhFGF-2, rhBMP-6, rhBMP-7, rhPDGF-BB, and VEGF189 (Figure 8A). A significant reduction in binding signal for each resulted from pre-treating the rhPln.D1 with heparinase III (Figure 8B) suggesting some GF affinity for HS. No binding with rhFGF-7 (Figure 8) or rhEGF, rhIGF, BMP-9, and VEGF165 (not shown) was detected by these methods. Results were similar using the rhPln.198 (not shown).

### Mitogenic activity of rhPln.247 and rhPln.198

**Figure 9:**
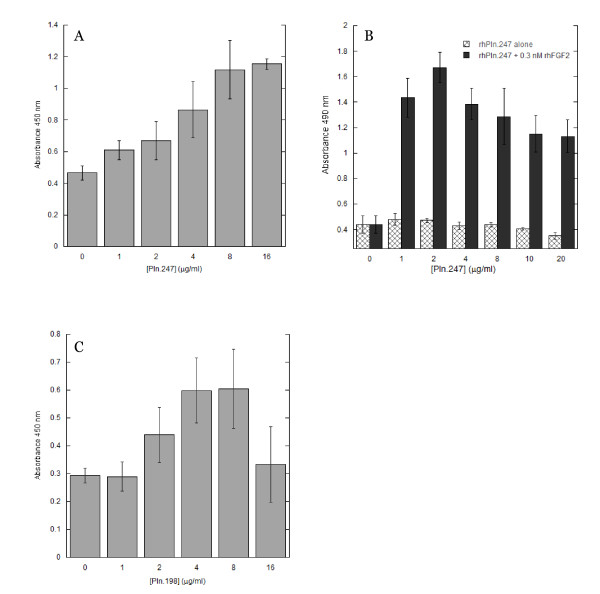
**Mitogenic Activity of rhPln.247 and rhPln.198**. HUVEC (panel A, C) or Baf32 cells (panel B) demonstrated a dose-dependent mitogenic response to rhPln.247 (panel A, B), or rhPln.198 (panel C). Panel B: Proliferation augmentation of FGFR1C expressing BAF32 cells by Pln.247 in the presence of 0.3 nM rhFGF-2 (solid). No proliferation enhancement was observed with Pln.247 without FGF-2 (crosshatch). In panel C, data represents the net absorbance for varying amounts of Pln.D1 with constant growth factor concentration after subtracting the contribution from Pln.D1 alone (without growth factor). Data are the average of four experiments.

A mitogenic dose response to rhPln.247 by HUVEC in low-serum growth medium occurred (Figure 9A). Using Baf32 cells, which have a limited mitogenic response to growth factors and express only the FGFR1c receptor, we demonstrated rhPln.247-dependant mitogenicity through the FGF-2/FGFR1c activation pathway (Figure 9B). Similar results were obtained with rhPln.198 also demonstrating a dose-dependent synergistic response of HUVEC to rhPln.198 in the presence of constant rhFGF-2 levels (Figure 9C).

## Discussion

Graham et al had demonstrated that a human perlecan domain 1 recombinant from HEK 293 cells was 67% HS, but that a fusion of domain 1 with the large C-terminal globular protein EGFP resulted in a 100% HS recombinant, similar to what is typically found in the native perlecan [31]. Also, Doege et al showed a predominately HS decoration of recombinant Pln.D1 when expressed with domains II and III in Cos-7 cells [37]. It appears, therefore, that structural changes to the C-terminus of domain 1 of perlecan can have a qualitative role in the post-translational glycosaminoglycan decoration of the core, and it is all the more interesting that the rhPln.198 and the rhPln.247, which are different by 49 amino acids at the C-terminus, were similar in their CS/HS ratio of approximately 2:1. Further, by gel analysis of glycosidase-treated recombinants (both rhPln.198 and rhPln.247) using both western blot (Figure 5), silver stain and Stains-All (not shown), recombinant was shown to be predominately synthesized with either only HS glycosylation or only CS glycosylation, but not with both, similar to the two pools of rhPln.D1 previously demonstrated using the mouse perlecan, one having only HS and the other containing CS [30,38].

It is postulated that the action of two separate enzymes dictates whether a beta-GalNAc or alpha-GlcNAc linkage is formed following the linker tetrasaccharide, hence initiating a CS or HS GAG, respectively (reviewed in [39]). In our work, separate pools of homogenous GAG types may have derived from variable, or more labile, tertiary structures of the truncated core proteins that favored either HS or CS enzymatic initiation. Alternatively, signals for specific HS or CS initiation, possibly relating to differential transit through the Golgi, were removed in truncation resulting in balanced populations of HS or CS species initiated in either the Golgi or trans-Golgi network, respectively [40]. Interestingly, the relatively higher proportion of CS-Pln.D1 in our enriched pools may be due to lower intravesicular pH, where more acidic vesicles are more likely to result in relatively less HS and relatively more CS [40]. Accordingly, the calculated pI of the rhPln.D1 is an acidic 4.5 as compared to a pI of 6.5 for the full length perlecan core.

These data demonstrated synthesis of recombinant Pln.D1 with highly heterogenous GAG chains contributing approximately 6-90 kDa to the rhPln.D1 core, consistent with the HS chain length previously estimated in rhPln.D1 characterized elsewhere [31] but greater than what had been estimated for recombinant mouse Pln.D1, also expressed in HEK 293 cells [30] or in CHO K1 cells [38]. In anion exchange chromatography, we hypothesized that recombinant Pln.D1 would elute primarily by degree of sulfation, though what we found was that the species with the lower net apparent kDa (shorter GAGs) eluted earliest. With increasing salt eluted rhPln.D1 species with increasing GAG length and greater apparent kDa. This would also suggest an overall similarity throughout each of the synthesized recombinant populations with regard to net sulfation.

Not only does the primary sequence of Pln.D1 permit both HS and CS [38], but the cell type in which Pln is produced can influence the GAG composition; mixed forms have been seen in EHS tumor cells [41], and glomerular epithelial cells [42]. Sulfation patterns on perlecan can also vary significantly depending on the cell type, and endothelial cells, including HUVEC, have previously been shown [43] to synthesize a perlecan with relatively less FGF-2 binding activity than other cell types, emphasizing the potential value of *in situ *post-translational modification with the use of recombinant biologics for therapy.

Typical interactions between FGF-2 and HS proteoglycans has been reported to range from k_D _= 0.5 nM for syndecan-3 [44] to k_D _= 2 nM for the secondary phase of binding to HUAEC perlecan [43], to k_D _= 15 nM for HS recovered from mouse embryonic fibroblasts [45]. Our estimated k_D _of 2 ± 0.2 nM for FGF-2 binding to both rhPln.198 and rhPln.247 synthesized by HEK 293 cells was similar in magnitude and virtually identical to the apparent net affinity of rhFGF-2 for a commercial HSPG. These data support the hypothesis that the quality of the HS decorating the rhPln.198 and rhPln.247 are quite similar.

While CS of rhPln.D1 has been shown to bind rhFGF-2 [33], in our system, chondroitinase ABC insignificantly affected the rhPln.198 or rhPln.247 binding to FGF-2. Our data suggested that HS participated in FGF-2 binding to the rhPln.D1 synthesized in HEK 293 cells while CS did not significantly contribute to the GF binding, similar to data reported for a pool of mouse Pln.D1 produced in a similar cell line [32]. Baf32 cells are an IL-3 dependent and HSPG deficient myeloid B cell line which have been stably transfected with either FGFR1c or FGFR3c [46,47]. The data presented here clearly demonstrated a strong co-receptor activity of the rhPln.247 with the rhFGF-2 signalling through the FGFR1c. Interestingly, mitogenic dose dependence of rhPln.247 in Baf32 cell culture supplemented with FGF-2 was also in the presence of low-level heparin. While the FGF-2/heparin combination provided no mitogenic effect, the FGF-2/rhPln.D1 combination in the presence of the heparin was highly mitogenic, highlighting the functional difference between the recombinant HSPG and heparin resulting from qualitative and quantitative differences in sulfation.

There is good evidence that the HS proteoglycans interact with several growth factors such as the VEGFs and BMPs via the HS chains [48-51]. Data presented support this potentially broad role of HS GAGs in mediating GF activities and support the possibility of enhanced growth factor function in wound healing through the use of recombinant HS proteoglycans.

Currently, no structural data exists for Pln.D1 and the limited sequence identity of Pln.D1 with other known structures causes difficulty in modelling of the full domain. The SEA domain, approximately 90 amino acids at the C-terminus of Pln.198, suggests a mixed α-helix and β-sheet configuration. The CD data presented for rhPln.198 and rhPln.247 confirm that hypothesis. rhPln.247 spectral analysis demonstrated a difference with rhPln.198 in the amount of helix vs. strand, which may be due to the 47 additional amino acids forming a C-terminal cysteine knot.

Assessment of the enriched recombinant pools using two different antibodies known to recognize domain 1 of perlecan (CSI 001-A71 and -A76) provided additional evidence that the proteoglycan pool was predominately recombinant D1 without detectable native D1. Support for this derives from published work showing mAb A76 to react relatively strongly with native perlecan D1, while reacting very weakly with a Pln.196-EGFP C-terminal fusion protein [52]. Further support was obtained in our labs with immunoblots demonstrating a significantly reduced binding signal from the A76 mAb relative to the A71 mAb for various rhPln.D1 species (not shown) and ELISA data showing poor A76 reactivity against rhPln.247 and strong reactivity with full-length endothelial perlecan, while A71 reacted strongly to both the native full-length perlecan and the truncated recombinant (inset Figure 2). Therefore, contaminating levels of native perlecan within the enriched recombinant preparations would have also been detected with the A76 antibody, but were not.

Pln.D1 plasmid expression has been used to generate relatively high levels of small, soluble, stable recombinant HS proteoglycan. While the benefits of inserting an alternative leader sequence have been described [31], we found that use of the CMV promoter with the native leader sequence had the potential for excellent expression of rhPln.198 and rhPln.247 from both the adenoviral and plasmid expressions systems, by both HEK cells and HUVECs. While we did not test constructs with the minimal native Kozak-like sequence for ribosome binding, we suspect that improvement of the Kozak sequence also contributed to a relative high expression level for these recombinant proteoglycan constructs.

## Conclusions

As more is presented and understood about proteoglycan synthetic pathways and their controlling factors, the applicability and usefulness of a secreted, functional, soluble, relatively small-sized recombinant HS proteoglycan should increase. With consistent synthesis and recovery of a HS-decorated rhPln.D1 pool that has the potential to bind and activate growth factors, the use of Pln.D1 as a research tool and therapeutic adjunct for a number of indications is possible and currently in testing.

## Authors' contributions

ALE carried out the infection/expression, purification, and circular dichroism while participating in the protein analysis gels and blots. ALE also carried out the ligand binding blots, enzymatic cleavage and blots, proliferation assays, and participated in the preparation of the manuscript. WP and GY carried out the cloning and adenoviral construction. KMJ contributed to the gels and blots and purified the virus for infection. CC performed the Baf32 proliferation experiment and native perlecan ELISA. JMW contributed to the ELISA data and protein purification procedures and participated in the manuscript preparation. AAD carried out the majority of the ELISA and oversaw all experimentation and manuscript preparation. All authors have read and approved the final manuscript.
